# Fracture of the Body of the Hamate With Dorsal Dislocation of the 4^th^ and 5^th^ Metacarpals: A Case Report

**DOI:** 10.2174/1874325001711010447

**Published:** 2017-05-30

**Authors:** Vasilis Athanasiou, Ilias D. Iliopoulos, Konstantinos Pantazis, Andreas Panagopoulos

**Affiliations:** Department of Hand Surgery, Orthopaedic Clinic of Patras University Hospital, Patras, Greece

**Keywords:** Missed injury, Hamate body, Fracture, Dorsal dislocation, Metacarpals, Internal fixation

## Abstract

**Background:**

Solitary fractures of the body of the hamate are rare. Their diagnosis is difficult and requires a high clinical suspicion and a proper radiological examination.

**Case report:**

We present a case of a 36-year-old male patient who sustained an intraarticular fracture of the body of the hamate along with dorsal dislocation of the 4^th^ and 5^th^ metacarpals on his right dominant hand. Through a dorsal surgical approach, he underwent ORIF of the hamate with screws and stabilization of the dislocated 4^th^ and 5^th^ metacarpals with KW. At his last follow-up appointment, 18 months postoperatively, he had no pain, almost full range of motion on his fingers and a Mayo Wrist score of 90 points.

**Conclusions:**

Hamate fractures are rare entities that can cause significant patient morbidity if not recognized and treated appropriately.

## INTRODUCTION

Fractures of the carpal hamate are not common injuries. These are estimated to occur in 2% - 4% of all carpal [bone fractures [1]. Coexistence with lesser metacarpals dislocation accounts for less than 1% of all hand trauma and is often referred to as fourth and fifth carpometacarpal (CMC) fracture-dislocation or as ring and small finger CMC injury [2, 3]. Mechanism of injury is usually a clenched fist strike against an unyielding object, however, indirect trauma may also be the cause. Diagnosis of such lesions can often be missed due to lack of familiarity with the injury and absence of obvious physical and radiological features [4]. There is no clear consensus over the management of acute CMC fracture-dislocations, as both conservative and operative methods have been shown to produce good results [5]. However most authors agree that delayed cases should be treated with open reduction and internal fixation (ORIF) in order to restore anatomy, prevent secondary dislocation and achieve full functional grip [5, 6]. We present the case of a missed hamate fracture associated with fourth and fifth CMC dislocation, which was treated with screw fixation of the hamate and KW fixation of the metacarpals.

### CASE REPORT

A 36-year-old male was attended the Α & E department of our hospital with a painful and swollen right (dominant) hand and wrist. He reported immediate onset of pain after a fist strike on a metallic door 3 days ago, for which he sought medical advice in another health institution in proximity to his residency. Initial radiographs of his wrist were interpreted as normal by the attending physician and the patient was discharged with painkillers and elastic bandage.

**Fig. (1):**
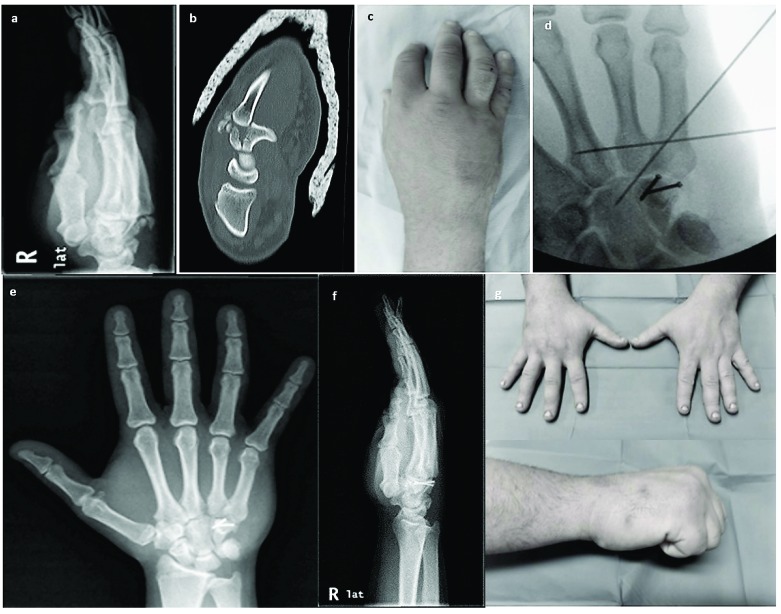
(**a**) Oblique 30o X-ray of the injured wrist showing dislocation of the 4th and 5th metacarpals. (**b**) CT scan showing the fracture of the hamate body, (**c**) clinical photo of the wrist showing diffuse swelling over the lesser metacarpals, (**d**) anteroposterior intraoperative (C-arm) X-ray showing fixation of the hamate with 2 small screws and reduction of the CMC dislocation with 2 KW, (**e**, **f**) anteroposterior and oblique X-rays of the wrist at 18 months showing healing of the hamate and congruent hamatometacarpal joint and (**g**) clinical photos of the wrist showing good range of motion and grip at the latest follow up.

Upon admission to our department, physical examination revealed massive swelling of the right hand and tenderness over the ulnar side of his right wrist, both palmar and dorsal. He had limited motion at the wrist, ring and little finger with pain during passive motion. The extremity was neurovascularly intact. Radiographic evaluation included x-rays (anteroposterior, lateral and oblique views) and computed tomography scan, which clearly demonstrated a coronal fracture of the body of the hamate associated with fourth and fifth CMC dorsal dislocation (Figs. **1a**-**c**). The patient underwent operative treatment at the day of presentation, consisting of ORIF of the hamate fracture and reduction of CMC dislocation with KW under image intensifier. Through a dorsal incision the hamate fracture was reduced and fixed with two small interfragmentary screws. The ring and little finger CMC dislocation was reduced applying longitudinal traction with a volar displacing force to the fourth and fifth metacarpals and 2 KW were driven to the third metacarpal and capitate bone (Fig. **1d**).

Post-operatively, the wrist was immobilized with a volar splint for 4 weeks with the hand elevated in a sling and no neurovascular deficit was recorded. The patient was discharged after dressing change and surgical wound inspection two days after admission.

Sutures were removed at 2 weeks follow-up in the outpatient department and at 6 weeks after the operation KW were also removed and the patient was given instructions on activity modification and physiotherapy. However, he failed to comply with medical instructions and at 3 months follow-up he admitted to have returned to heavy manual labor without attending any physiotherapy sessions. Nevertheless, the patient was very satisfied with treatment outcome reporting just a slight discomfort when lifting heavy objects. At his last follow-up appointment 18 months post-surgery, the patient was asymptomatic with almost full range of wrist and finger motion and a Mayo wrist score of 90 points (Figs. **1e**-**g**).

## DISCUSSION

The hamate bone is wedge shaped and has a hook like process, the hamulus. The proximal part articulates mainly with triquetrum and the apical proximal part of the wedge with lunate. Laterally, it articulates with capitate and distally with the base of the fourth and fifth metacarpal. Hamate fractures are rare injuries and according to Milch’s classification there are two subtypes: fractures of the hook (type I) and fractures of the body (type II), these fractures were considered stable with no need for operative treatment [7]. However, Ebraheim *et al.* [8] based on cadaveric studies, described 3 types of coronal hamate body fractures, which can result in highly unstable injuries associated with fifth and fourth CMC dislocation. These lesions usually require ORIF but were considered very rare to be included in Milch's classification.

The hamatometacarpal articulation is a saddle joint with a convex base of the 5^th^ metacarpal fitting into a concave facet on the hamate. Both bones have an additional flat facet for articulation with the 4^th^ metacarpal. The hamatometacarpal joint is connected by strong volar, dorsal and interosseous ligaments. Stability is further reinforced by broad insertions of the wrist flexors and extensors, though, dorsal dislocation is prevented only by the dorsal ligament [6]. Coronal fractures of the hamate have been shown to occur when a force is transmitted longitudinally along the lesser metacarpals (4^th^, 5^th^) with the wrist in ulnar deviation as, in a neutral position, the same force would most probably cause a boxer’s fracture [9, 10]. Additionally, palmar flexion of the wrist at the moment of impact will result in the dorsal displacement of the involved metacarpals due to the obliquity of the fifth CMC joint and the pull of extensor carpi ulnaris (ECU) and flexor carpi ulnaris (FCU) tendons along with the hypothenar muscles [11].

Cain *et al.* [2] classified hamatometacarpal fracture-dislocation (HMFD) into 3 types based on hamate fragmentation but with several limitations. Their classification scheme was confined to a prerequisite fourth metacarpal base fracture, while complex intrarticular fracture patterns could be interpreted due to the limitations of plain radiography. Kim *et al.* [12] in a recent study of 21 patients with a HMFD, proposed a different classification for these injuries based on preoperative computed tomography (CT): type I consists of a simple dislocation while in type II there is an associated fracture, either at the base of the 4^th^ metacarpal (subtype A) or the hamate’s articular surface (less than one third – subtype B). In type III, in which 11 out of 21 patents of the study were categorized, there is a dorsal hamate fragment of more than one-third of the articular surface. Kim recommends conservative treatment for type I lesions, percutaneous KW fixation for type II and ORIF for type III. However this treatment algorithm does not distinguish between acute and delayed cases.

Hamatometacarpal fracture-dislocation may be missed at initial presentation up to 71% in some studies [13]. Pain and swelling are the main clinical findings but the rarity of this traumatic event along with subtle deformity in some cases, may cause it to go unnoticed [1, 14]. Moreover, routine radiographic evaluation with anteroposterior and lateral views of the wrist may not reveal the lesion, being only visible with an oblique view of 30° of forearm pronation [15]. Nevertheless, a high resolution CT scan is considered mandatory to completely evaluate these injuries and decide on treatment plan [5, 12, 14, 16].

Different treatment options have been proposed in the literature to address these lesions and decision over operative or conservative approach for the simple types remains controversial [5, 9, 14]. Conservative treatment has been proposed for acute cases with very good results but, in the setting of late presentation, closed reduction seems to be inadequate and lead to poor outcome [5, 6]. Zhang *et al.* [5] reported on 26 patients with fourth and fifth CMC fracture-dislocation up to a follow-up of 1 year. They reported excellent functional outcome for 20 acute cases treated conservatively but noticeable deformity, pain and a poor functional result for 3 out of 6 delayed cases managed non-operatively. Closed reduction and percutaneous KW fixation has also been reported in the literature but with inferior results comparing to ORIF in the setting of displaced fractures [17, 18]. Wharton *et al.* [18] used this method to treat 14 patients with coronal hamate fractures and reported incomplete reduction and less favorable results for patterns with displacement.

Open reduction and internal fixation seems to be preferred over conservative treatment or closed reduction and percutaneous pinning, especially for delayed cases and large displaced fragments of the hamate bone [1, 3, 9, 14, 19-22]. Anatomic reduction and congruent stability of the CMC arch is mandatory to achieve a good result. Finally, Carrico *et al.* [21] in their recent case report of a complex coronal hamate fracture and fifth CMC dislocation, recommended the use of a temporary spanning external fixator for 6 weeks in order to maintain reduction of the little finger CMC joint [21].

### CONCLUSION AND TAKE HOME MESSAGE

Coronal hamate fracture-dislocations are rare injuries with a high rate of missed initial diagnosis. A high degree of suspicion is needed to reveal the lesion. When a traumatic event is associated with proper clinical examination, an oblique x-ray of the wrist must be obtained followed by CT confirmation and further evaluation of fracture morphology to allow optimal surgical planning. In most cases, these injuries should be managed as intrarticular fractures with ORIF in order to achieve anatomic and stable reduction and allow for early mobilization and good functional outcome.

## LIST OF ABBREVIATIONS

CMC: = Carpo Meta Carpal
ECU: = Extensor Carpi Ulnaris
FCU: = Flexor Carpi Ulnaris
HMFD: = Hamato Metacarpal Fracture Dislocation
KW: = Kirschner Wire
ORIF: = Open Reduction Internal Fixation
